# Pharmacist-Initiated Medication Error-Reporting and Monitoring Programme in a Developing Country Scenario

**DOI:** 10.3390/pharmacy6040133

**Published:** 2018-12-14

**Authors:** Sri Harsha Chalasani, Madhan Ramesh, Parthasarathi Gurumurthy

**Affiliations:** 1 Department of Pharmacy Practice, JSS College of Pharmacy, JSS Academy of Higher Education and Research, Mysuru 570015, Karnataka, India; csriharsha@live.com; 2 Dean—Global Engagement, JSS Academy of Higher Education and Research, Mysuru 570015, Karnataka, India; partha18@gmail.com

**Keywords:** medication errors, clinical pharmacist, patient safety, medication incidents

## Abstract

Medication errors (MEs) often prelude guilt and fear in health care professionals (HCPs), thereby resulting in under-reporting and further compromising patient safety. To improve patient safety, we conducted a study on the implementation of a voluntary medication error-reporting and monitoring programme. The ME reporting system was established using the principles based on prospective, voluntary, open, anonymous, and stand-alone surveillance in a tertiary care teaching hospital located in South India. A prospective observational study was carried out for three years and a voluntary Medication Error-reporting Form was developed to report medication errors MEs that had occurred in patients of either sex were included in the study, and the reporters were given the choice to remain anonymous. The analysis was carried out and discussed with HCPs to minimise the recurrence. A total of 1310 medication errors were reported among 20,256 hospitalised patients and the incidence was 6.4%. Common aetiologies were administration errors [501 (38.2%)], followed by prescribing and transcribing errors [363 (28%)]. Root-cause of these MEs were distractions, workload, and communications. Analgesics/antipyretics (19.4%) and antibiotics (15.7%) were the most commonly implicated classes of medications. A clinical pharmacist initiated non-punitive anonymous ME reporting system could improve patient safety.

## 1. Introduction

Patient safety is at the core of quality of health care as echoed in the Hippocratic Oath: “I will prescribe regimes for the good of my patient according to my ability and my judgment and never do harm to anyone … In every house whenever I come, I will enter only for the good of my patient” (Excerpt from the Hippocratic Oath c. 300–400 BCE.) The Hippocratic Oath guided most doctors to be non-maleficence and beneficence for a long time [1]. Unfortunately, these virtues in newer times are troubled by the aged predators called Medication Errors [2]. 

Despite of the growing attention to patient safety, the freedom from accidental injury due to medical care or from medical error, the realisation of this apophthegm is far from long shot [3,4,5]. Working on Patient safety has stuck a realisation that the power and complexity of modern medicine to cure and ameliorate ailments rendered hospitals as a ‘not so safe place’ for healing as perceived yet, those were places fraught with risk of patient harm [6]. 

Long before the words “medication error” and “patient safety” came into existence, the term Iatrogenesis had been widely known amongst the medical fraternity. This ancient word, iatrogenesis, is a combination of two Greek words, Iatros (healer) and Genesis (to bring forth). Iatrogenesis is defined as the undesirable side-effects of medical interventions [7]. Whereas, National Coordinating Council for Medication Error-reporting Programme (NCC MERP) defines medication error as “A medication error is any preventable event that may cause or lead to inappropriate medication use or patient harm while the medication is in the control of the health care professional, patient, or consumer. Such events may be related to professional practice, health care products, procedures, and systems, including prescribing, order communication, product labelling, packaging, and nomenclature, compounding, dispensing, distribution, administration, education, monitoring, and use. While near misses are defined as an event, situation, or error that took place but was captured before reaching the patient” [8,9].

Medication Errors, once known as a diseases of medical progress [10] in the 1950s and a ‘noxious episodes’ [11] in 1960s, evolved into a vast, profound, inevitable, and ineluctable reality of medication-based therapy. The cause of this evolution was the repercussions of current medicalisation of diseases and had dreadful effect on patients and medical staff alike [12]. Although hospitals and healthcare professionals (HCPs) avow and aim for providing the safest care possible, things may go wrong leading to extensive toll of inadvertent clinical iatrogenesis. 

In 1984, the Harvard Medical Practice Study (HMPS) findings sparked the need for replication of HMPS in Australia, New Zealand, Denmark, the United Kingdom, and Canada, and altogether reported higher rates of adverse events (9–13%) and, roughly 50% of the adverse events were considered preventable [13,14,15,16,17]. The consecutive evidences of medication errors recorded in that decade transcended into the wake of sensational report by the Institute of Medicine (IOM), Building a Safer Health System, November 1999. This report has changed all the existing estimates of medication errors and claimed as many as 98,000 people die every year because of iatrogenesis in the United States of America (USA) alone. Followed by the United Kingdom (UK) Department of Health’s review of patient safety in the National Health Service (NHS), 2000, ‘An organisation with a memory’, extrapolated iatrogenic figures from the US studies to estimate that 250,000 patients might be iatrogenic suffering as a result of NHS care costing around £400 million per year, and the additional hospital bed days cost as much as £2 billion annually [18].

By 2013, various researchers who provided an estimate of 210,000 deaths per year were associated with preventable adverse events (PAEs) in hospitals using the Global Trigger Tool (GTT) [19]. These estimates were the lower limits of weighted averages of the various contemporary studies. With incomplete medical records these proposed values can be projected to an estimate of 400,000 per year premature deaths associated with preventable harm. Further, serious harm seems to range 10- to 20-fold more common than lethal [20]. 

On the other hand, these estimates of medication errors are inundated for not being absolute as it is pragmatically difficult to compute and arrive at prevalence rate of the errors due to the varying definitions of medication errors and classification systems. Usually, the reported rates vary depending on the denominator used (e.g., number of patients, number of prescriptions, number of doses or a specific medication etc.). The challenge of reaching a nearest possible estimate is compounded by differences in health care system organizations and the availability and use of medication error-reporting systems [21]. Whilst in the Indian scenario, the current workload on doctors (1:1800) and nursing staff (1.7 for every 1000 patients) has left breaches in patient safety [22]. Recently, blotched mass sterilisations and cataract surgeries in 2014 remain stark are reminders of inadequate accountability, limited infrastructure, and low-quality health services in India’s health care sector [23]. 

With this scenario, sustaining a well-established medication error-reporting system is an uphill battle in any developing economic state. Although there is a lack of medication error-reporting system in India, the few studies that have been conducted on medication errors and rate of errors ranged between 15.34% and 25.7%, respectively, in hospitalized patients [24,25]. The confidence in these numbers suffer due to severe under reporting and lack of uniform standards in gauzing the errors [26]. This scenario warrants that designing an indigenous error-reporting programme is essential to understand the nature and extent of medication errors to encourage patient safety.

## 2. Methodology

At the study site, the Dept. of Clinical Pharmacy has taken an initiative to design and implement medication error-reporting and a monitoring programme using the principles of prospective, voluntary, open, anonymous, and stand-alone surveillance in an 1800-bed tertiary care teaching hospital located in South India. Further to support the system a five-membered expert panel was created to oversee its functions. Members were drawn from various specialities within the hospital and were set to meet once every two months or, otherwise, on the basis of need to evaluate, monitor, and redress the medication errors and any other issues pertaining to the patient safety. NCC MERP’s definition of medication error was adapted for this study purpose and any deviation from the standard procedure involved in the prescribing, transcribing, dispensing, administration, and monitoring were considered as medication errors. The elements of the deviation are tabulated in Table 1 of results.

For the purpose of the study, a prospective, observational study design was adapted and carried out for three years. MEs involving in-patients of either sex admitted to the wards of General Medicine, Emergency Medicine, Intensive care units, Surgery, and Obstetrics and Gynaecology were included in the study. The reporters were given the choice to remain anonymous and Institutional human ethics committee approval was obtained to carry out the study.

The functional aspects of the medication error-reporting system include:

- *Voluntary*: Health care professionals can report the medication errors without any compulsion.
- *Open*: Any health care professional of any specialisation of any status can report any medication error.
- *Anonymous*: Health care professionals can remain anonymous while reporting the medication error.
- *Stand-alone*: Thus, reported medication errors were pooled into a database and the data was secured and not shared outside the purview of the review committee.
- *Exploratory*: Reported medication errors were evaluated with the concept of “cause and effect”
- *Paper and electronic based database*: Reporter can use either of the available modes to report an error. HCPs were requested to avoid reporting the same medication error by using two different modes.

Medication Error reports were accepted from all the health care professionals of the different specialities irrespective of their status and types of services they provided. As mentioned earlier, the reporters were not obligated to disclose their identity while reporting. They were requested to report the error with a detailed description using any of the provided modes of reporting. For the study purpose, a system was created wherein a reporter can choose to report the error using paper-based system, electronic system and or telephone-based system.

### 2.1. Paper-Based System

The Voluntary Medication Error-reporting Form (vMERF) was designed by clinical pharmacists after observing the MedWatch^TM^, Institute for Safe Medication Practices (ISMP), ISMP-Canada, National Co-ordination Centre Medication Error-reporting Programme (NCC MERP), Joint Commission: Accreditation, Health Care, Certification (JCAHO) sentinel event reporting programs. Draft voluntary medication error-reporting form was circulated to all health care professionals for their feedback, and suggestions on utilisation of the vMERF were considered and incorporated as appropriate.

The final vMERF includes following elements:

- Medication Error’s date and time
- Patient’s age and gender
- Description of the medication error
- At which stage the medication error has occurred
- Whether the medication error has reached the patient, and if reached, did the error warranted for any intervention
- Personnel who identified and reported the medication error
- Place where the medication error has occurred
- NCC MERP’s medication error outcome category
- Contributing factors
- Details of the medication involved
- Optional details of the reporter.

### 2.2. Electronic and Telephonic Reporting System

To make reporting the ME simplified, an intranet system was developed by the clinical pharmacists and incorporated into the hospital information system (HIS) portal, wherein any HCP can log into their HIS portal account and can report a ME. The electronic form contains same elements as that the of paper form to report an error. The committee members can view and respond to these errors spontaneously. For instant reporting and immediate attention, a telephonic system was created. The speed dial telephone numbers of the expert panel and the clinical pharmacists were widely publicised in the hospital. Any HCP who wishes to use this system can dial the assigned numbers throughout the day and report an error. Use of either system could compromise the reporter’s anonymity and this was a limitation.

### 2.3. Implementation of the System

To create awareness on the importance of reporting the MEs and encourage spontaneous reporting by health care professionals, “Dear Health Care Professional” letters were drafted by the clinical pharmacists under the guidance of expert panel and distributed amongst all HCPs. Patient safety classes were conducted to the HCPs by the clinical pharmacists wherein the expert committee’s feedback letters were provided to the health care professionals. Further, these classes were liaised with the interdisciplinary members of the hospital and aimed at emphasising the need for reporting of medication errors without any fear.

### 2.4. Collection of Reports

Drop boxes were made available at all nursing stations for easy access to paper vMERFs along with the facility to drop the filled forms. These forms were collected every 24 h. When a medication error report was dropped in one of the drop boxes, a clinical pharmacist would personally collect the form and follow up with the concerned HCPs to collect supportive information on what factors were involved in this error, how best the error could have been avoided, and any missing information in the report. Wherever possible, appropriate data pertaining to the reported errors was collected from the various data sources such as patient’s case file, interaction with other health care professionals, patients and their care providers etc. The reporters themselves were asked to mention the contributing factors involved in the error, such as work load, distractions, newer staff, ambiguous communications etc.; thus, the reported contributing factors of the respective medication errors were rather subjective but not quantified, which was another limitation.

### 2.5. Assessment of the Reports

**Figure 1:**
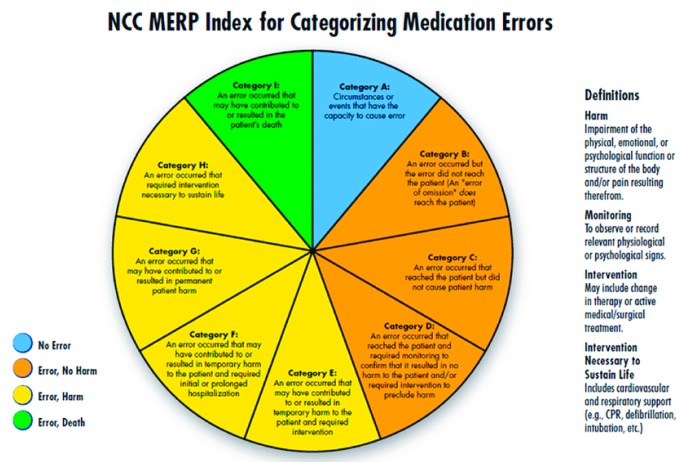
National Coordination Centre Medication Error Reporting Programme (NCC MERP) Index for Categorising Medication Errors.

During the analysis, a clinical pharmacists presented each of the reported error to the panel regularly twice a week to understand how an error has occurred. Their pattern, contributing factors, and root causes were closely monitored. NCC MERP’s Medication Error Index, which classifies an error according to the severity of the outcome was adopted to assess the severity of the reported errors. According to the index, the outcomes were classified from Category A to Category I (Figure 1). Based on these findings, medication error preventive strategies were developed accordingly, e.g., high risk medication tags to caution the end user, separation of look-alike and sound-alike drugs and train pharmacists etc. Data analysis was done using descriptive statistics. 

Wherever the patient identifiers were available, they were followed-up until discharge to ensure that the medication error has been addressed appropriately. Identifier details of the patient and other information related to the error were kept confidential.

## 3. Results

A total of 1310 errors were reported; the highest number of medication errors occurred during the medication administration stage [501 (38.2%)], followed by prescription errors [243 (18.5%)], dispensing errors [223 (17%)], procurement related errors [223 (17.02%)], and lastly, transcription errors [120 (1.29%)] were reported.

The majority of the medication errors were reported from the department of emergency medicine [458 (35%)] followed by Surgery and Obstetrics and Gynaecology specialities [304 (23%)]. Nonchalant operational conditions have resulted in the fewer medication errors [256 (19%)] in general medicine wards. During the study period, clinical pharmacists reported the highest medication errors [674 (51%)], followed by nursing staff and doctors [409 (31%)] and [227 (17%)] respectively.

The majority of the reported medication errors belonged to Category A [432, (33%)], where the circumstances were potential to cause an error, while the least belonged to the Category F [1 (0.07%)]. None of the reported medication errors had outcomes corresponding to the Category G and above. These results were represented in Graph 1.

**Graph 1.** NCC MERP’s categorizing index of the reported medication errors.


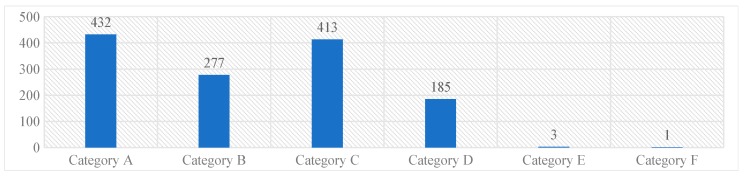


Work shifts were classified as work shift 1 (08:00–14:00 h), 2 (14:00 to 20:00 h), and 3 (20:00 h–06:00 h), wherein, majority of medication errors were reported during third work-shift [425 (32%)], while the fewer were reported during first work-shift [407 (31%)]. Over all, the difference in medication errors reported during three work shifts was limited. Graph 2 represents the distribution of errors over different work-shifts.

**Graph 2.** Distribution of medication errors occurred over different work-shifts.


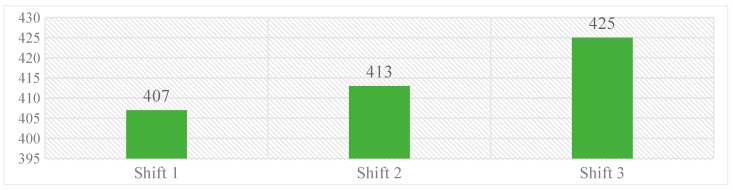


Our study reveals that the highest medication errors occurred due to distractions during work (473), while the lowest were due to calculation errors (55). Graph 3 reflects the distribution of various contribution factors causing the medication errors.

**Graph 3.** Distribution of contributing factors of medication errors.


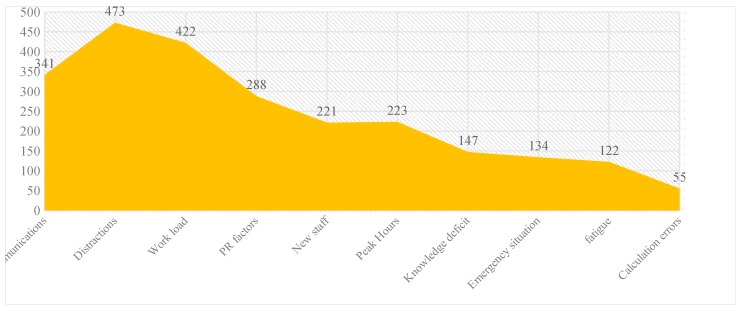


Of the medicines implicated in the medication error reports, antipyretics/analgesics [255 (19.46%)] were the main ones, followed by antibiotics [206 (15.72)]. The detailed distribution of the medicines implicated are tabulated in Table 2.

## 4. Discussion

The incidence of MEs at the study site was 6.4%. While, in India, the incidence of medication errors ranged between 3% and 33.4% [27,28]. Such a broad range in the ME rates may be attributable to the disparities in the methodologies adopted in identifying and reporting the errors and its associated variables such as definition of medication error adapted, type of the medication error-reporting implemented, nature of the hospital, i.e., workforce and work load, occupancy ratio, etc., duration of the study and type of the patients followed [29,30,31,32]. 

Enforcing a mandatory reporting system may lead to coercing the covert of reporting medication errors within the HCPs. 

On the other side, voluntary reporting may not be absolute as HCP’s intellectual domains on overall awareness on MEs and what, how and where to report, and the ability to handle the repercussions of reported errors without resorting to the blame culture influences the overall reporting trends. Hence, many errors remain under-reported. In Barach et al.’s study, the under-reporting of MEs is estimated to vary from 50% to 60% annually and reporting is usually done in an informal manner. Although, errors are usually discussed verbally at morbidity or mortality review meetings without formal written reports [33]. Implementation of medication safety practices and improvement of patient safety domains remains a mirage san written reports.

Across the globe, the incidence of medication errors is almost certainly low and not often reliable due to under-reporting tendencies. Reasons for underreporting may span across apprehension of reprisal, lawsuit concerns, time constraints, the uncertainty of which errors to report, concerns of implicating other HCPs, peer pressures, and lack of feedback on reported MEs [34,35]. Further, the information on incidences of medication errors that are reported in selected clinical specialisations with focus on certain medications were well documented; however, the overall data pertaining to the incidence of medication errors occurring in a complete hospital setup like ours is limited. Despite detecting and reporting the medication errors, quantifying these errors’ rates is not an easy task. Anonymous reporting may encourage the reporters to report an error, but addressing patient safety under the anonymous tag may get compromised by repetitive and preventable errors [36]. Further, enormous diversity in the taxonomy used in reporting medication errors, and expressing the error rates using various denominators such as patient days, for 100 prescription, for 100 doses, etc., is an arduous task in harmonising the overall error rate [37].

### 4.1. Overall Trend in Voluntary Reporting of Medication Errors

All medication errors were reported using a paper-based system as it may relatively protect the anonymity of the reporter compared to electronic and telephonic systems. The doctors, nursing staff, and clinical pharmacists reported 227 (17.3%), 409 (31%) and 674 (51.4%) errors, respectively, in five selected different study units. Of these errors, doctors reported medication administration errors [72 (31%)], majorly followed by prescription errors [49 (22%)], and dispensing errors [31 (14%)] and so on. Amongst the errors reported by the nursing staff, the majority were procurement errors [89 (22%)] followed by transcription and administration errors [70 (17%)] each. Lastly, clinical pharmacists reported administration errors [211 [n = 674 (31%)] followed by dispensing 138 (21%) and prescription errors [129 (19%)]. In our study setting, clinical pharmacists reported the majority of the medication errors compared to other health care professionals for two reasons. Firstly, the amount of time CPs spend with their patients in the wards and secondly, the reporting system is driven by CPs themselves.

Further, this reporting system is the first of its kind in our setting, and factors such as availability of the time for HCPs for reporting an error, their workload, fear of legal liability, misconceptions on job threat, adverse economic effects, concerns regarding protecting the professional status at the workplace, and other negative consequences of reporting might have played an important role on why there were fewer errors reported by the doctors and nursing staff [38]. In order to understand and overcome these factors, one-to-one and group discussions were conducted periodically by the expert committee, reinforcing the non-punitive and anonymous principles of the reporting system.

Another reason for lower medication error-reporting rate compared to the earlier mentioned studies, which was we identified during casual discussions with health care professionals, was “preference” of doctors and nursing staff, to keep medication errors in-house. Wherein, believing loyalty to colleagues, and “whistle-blowing” were both unsupportive and unethical. Similar opinions were quoted in age old Webb et al.’s study [38]. Another reason for lower reporting of Medication errors is that, they often instigate remorse in the implicated personnel. Adapting a mandatory reporting system will drastically affect the overall reporting trends and having a voluntary reporting system is by no means an antidote for the complacency attitude amongst HCPs. We presume, the extent of such barriers may influence the reporting rates across the developing countries.

One of the strategies to reduce the impact of such preferences on overall reporting rate is utilisation of clinical phatrmacists in medication error-reporting systems. They can support the HCPs by maintaining the confidentiality of errors, obtaining detailed medication history from the patients and their care takers and provide efficient medication reconciliation, and thus reducing the medication errors [39,40]. The utilisation of the clinical pharmacists in the current study set-up has been the reason for reporting the majority of the errors and, thus, reducing associated implications.

### 4.2. Outcomes of the Reported Errors

Most of the medication errors were reported to have the potential to cause error [432, (32.9%)]. Followed by errors that occurred and did not reach the patient [413 (31.5%)]. These errors were intercepted by the clinical pharmacist during the ward rounds and intensive follow-up of the patients and prevented them from manifesting into actual errors. Category A errors are the errors which have the potential to cause an error, and Category B errors are actual errors which were identified and prevented before reaching the patient. Distractions while filling the prescriptions and drawing the medicine into syringes, illegible hand writing in prescriptions etc., have potential to cause an error if not rectified immediately. Further, when the Category A errors are left unattended, they may evolve into Category B level errors, that are just might reach the patient if not stopped by the HCPs. From there onwards, the progress of the category of these errors depends on to what level the patient was affected. The range of such errors is confined between Category C and I, wherein category C medication errors are those that have reached the patient but did not affect safety, and category I errors might have caused patient death. 

Employing double checks on medication usage at every step by those whoever are responsible in handling the drugs can reduce medication errors effectively than any sheer luck interceptions. As clinical pharmacists are bestowed with more patient bed-side time, they intercepted the potential errors and reported. Implementation of the Computerised Physician Order Entry systems (CPOE) could reduce the progression of errors from Category A to Category I. However, use of such systems is modest in the resource restricted countries. Towards inculcation of patient safety culture amongst all HCPs, we encouraged them to report voluntarily whatever they deemed an error irrespective of their severity. This has encouraged the HCPs to report Medication Errors. Giving incentives to the HCPs could improve the overall rate of ME reporting, but it may not be viable model in the developing countries. 

### 4.3. Contributing Factors

Distractions were one of the major contributing factors for reported medication errors followed by increased workload and incoherent communications. Being a 1800-bed hospital with a constant 80% occupancy rate and housing hundreds of HCPs, the contributing factors such as distractions and work load are likely to be more. A total of 2560 contributing factors were associated with reported 1310 medication errors (a medication error can have multiple contributing factors). 

Further, we have identified that the majority of the medication errors were reported during night shifts 425, [(32.4%) n = 1310] (20:00 to 08:00 h) followed by 407, [(31.06%) n = 1310] during the first shift (08:00 to 14:00 h). The reporting rate of medication errors during night seems to be lesser compared to other shifts. HCPs regularly sacrifice their breaks to provide patient care, therefore during the night shifts bright lights, rest breaks, power naps, and exercises, can be used to provide relief from the symptoms of fatigue. To reduce distractions and improve interpersonal communications, CPOE systems can be employed and thus improving patient safety [41].

## 5. Conclusions

As a considerable number of MEs were preventable, it is important to develop and implement strategies that are unique to the work environment to try to overcome such MEs in the future. Intense monitoring of patients for potential MEs and its early detection and reporting by all HCPs may result in improved therapeutic outcomes and decreased unnecessary healthcare related mortality. 

Medication errors, such as the ones reported here, not only harm the patients, but also wreck the confidence of HCPs involved. Reporting systems should be designed to encourage and instil the confidence in reporting the errors openly by HCPs. Our study laid foundations for successful establishment of medication error-reporting and monitoring programme in a tertiary care teaching hospital and is contributing significantly towards the patient safety involving all stakeholders. With greater emphasis on a non-punitive course of corrective measures and anonymity of the HCPs on handling medication errors, improves the overall reporting in terms of quality and quantity. Fault finding attitudes should be replaced with open, inclusive, and common improvement agendas involving all HCPs alike for fortifying patient safety. Clinical pharmacists, working in the close liaison with other HCPs can provide excellent support in promoting and sustaining patient safety. 

This study is indeed a pilot study to understand the implications of Medication Errors at our study site. The three years have given us deeper insights into how HCPs react to the varying levels of MEs. We promptly discussed the MEs with concerned HCPs on the ethical basis to alert them regarding such happenings. HCPs intervened on time to minimise the impact of these errors. This learning experience has helped all HCPs to be cautious of such happenings in the future, and they emphasised the importance of prevention of errors to the associated team members. In this way, this study has contributed to the early steps towards patient safety. Overall, this is a first of its kind study in our region and we are trying to balance the interests of all stakeholders, most importantly, the patient’s.

## Figures and Tables

**Table 1:** Detailed distribution of Medication errors across various stages.

Sl. No.	Level	Type of Error Identified		No. of Errors (%) (*n* = 1310)
1	Prescription	Date and time missing		9 (0.6)
		Patient details incomplete (name, age, and gender)		33 (2.5)
		Order is not in CAPS		31 (2.3)
		Unclear instructions		14 (1.06)
		Dose missing		71 (5.4)
		Quantity missing		09 (0.68)
		Frequency missing		37 (2.82)
		Signature of prescriber missing		08 (0.61)
		Seal of the prescriber missing		14 (1.06)
		Details of prescriber missing		17 (1.29)
Total				243 (18.5)
2	Transcriptional	Date and time missing		13 (0.99)
		Patient details incomplete		17 (1.2)
		Order is not in CAPS		19 (1.4)
		Unclear instructions		01(0.07)
		Dose missing		32 (2.44)
		Quantity (details missing/incorrect)		04(0.30)
		Frequency (details missing/incorrect)		07 (0.53)
		Frequency details missing		07 (0.53)
		Overwriting		03 (0.22)
		Signature and seal of transcriber missing		17 (1.29)
Total				120 (9.1)
3	Dispensing	Wrong Drug		58 (4.42)
		Wrong strength	Greater	28 (2.13)
			Lesser	22 (1.6)
		Wrong dosage form		17 (1.29)
		Wrong Quantity	Fewer	15 (1.14)
			More	23 (1.75)
		Substituted with another brand		22 (1.67)
		Delayed Dispensing		38 (2.9)
Total				223 (17.0)
4	Administration	Wrong Drug		22 (1.67)
		Wrong strength	Greater	39 (2.97)
			Lesser	16 (1.22)
		Wrong dosage form		03 (0.22)
		Wrong time	Delayed beyond 1 h	68 (5.19)
			Administered before 1 h	28 (2.13)
		Wrong administration technique		10 (0.76)
		Wrong patient		03 (0.22)
		Wrong route of administration		70 (5.34)
		Dose missed		94 (7.17)
		Document-ation	Incomplete documentation	15 (1.14)
			Delayed Documentation	84 (6.41)
		Monitoring		34 (2.59)
		Storage		15 (1.14)
Total				501 (38.2)
5	Procurement	Fail to bring		96 (7.32)
		Insufficient Quantities		53 (4.04)
		Substituted with other brands		22 (1.67)
		Time taken to procure is beyond 12 h		52 (3.96)
Total				223 (17.02)
Grand Total				1310 (100)

**Table 2:** Classification of medicines involved in reported medication errors.

Class of the Drugs	Drugs	No. of Errors (%) (N = 1310)		
Analgesics/Antipyretics	Aceclofenac	M01AB16	23 (1.75)	
	Aspirin	B01AC06	56 (4.27)	
	Diclofenac	M01AB05	17 (1.29)	
	Gabapentin	N03AX12	16 (1.22)	
	Ibuprofen	M01AE01	08 (0.61)	
	Indomethacin	M01AB01	06 (0.45)	
	Mefenamic Acid	M01AG01	28 (2.13)	
	Naproxen	M01AE02	03 (0.22)	
	Paracetamol		N02BE01	61 (4.65)
	Piroxicam	M01AC01	01 (0.07)	
	Pregabalin		N03AX16	21 (1.60)
	Tramadol	N02AX02	15 (1.14)	
Total				255 (19.46)
Anti-Arrhythmic drugs	Digoxin		C01AA05	3 (0.22)
Antibiotics	Beta-Lactam	Amoxicillin + Clavulanic Acid	J01CR02	16 (1.22)
	Cefixime +Clavulanic Acid		J01DD08	01(0.07)
	Cefotaxime		J01DD01	12 (0.91)
	Ceftriaxone		J01DD04	07 (0.53)
	Cefuroxime		J01DC02	03 (0.22)
	Ciprofloxacin		J01MA02	13 (0.91)
		Crystalline Penicillin	J01C	16 (1.22)
Quinolones	Norfloxacin		J01MA06	13 (0.99)
Glycopeptide	Vancomycin		J01XA01	11 (0.83)
Oxazolidinone	Linezolid		J01XX08	19 (1.45)
Macrolides	Azithromycin		J01FA10	64 (4.88)
Aminoglycosides	Gentamicin		J01GB03	12 (0.91)
	Amikacin		J01GB06	11(0.83)
Nitroimidazoles	Metronidazole		J01XD01	03 (0.22)
Total	206 (15.72)			
Antidiabetic	Acarbose		A10BF01	8 (0.61)
	Glimepiride		A10BB12	32 (2.44)
	Glyburide		A10BB01	17 (1.29)
	Insulin		A10AC01	43 (3.28)
	Metformin		A10BA02	56 (4.37)
	Voglibose		A10BF03	1 (0.07)
Total				157 (11.98)
Anti-Emetic	Domperidone	A03FA03	81 (6.25)	
	Doxylamine Succinate		R06AA09	02 (0.15)
	Itopride		A03FA07	01 (0.07)
	Metoclopramide		A03FA01	13 (0.99)
	Ondansetron		A04AA01	12 (0.91)
	Promethazine		R06AD02	01 (0.07)
Total				110 (8.39)
Anti-Epileptics	Clobazam		N05BA09	1 (0.07)
	Diazepam		N05BA01	1 (0.07)
	Phenobarbital		N03AA02	1 (0.07)
	Phenytoin		N03AB02	3 (0.22)
Total				06 (0.45)
Anti-Hypertensive	Atenolol		C07AB03	21 (1.6)
	Enalapril		C09AA02	04 (0.30)
	Metoprolol		C07AB02	34 (2.5)
	Propranolol		C07AA05	19 (1.45)
Total				78 (5.95)
Anti-Secretory	Omeprazole	A02BC01	16 (1.22)	
	Pantoprazole		A02BC02	122 (9.31)
	Rabeprazole	A02BC04	21 (1.60)	
Total	159 (12.13)			
Anti-Tussive	Ambroxol (Secretolytic)		R05CB06	24 (1.83)
	Dextromethorphan		R05DA09	13 (0.99)
Total				37 (2.82)
Antiviral	Acyclovir		J05AB01	2 (0.15)
B_2-_ Agonist + Anti-Cholinergic	Salbutamol + Ipratropium	R03AL02	127 (9.69)	
Total	127 (9.69)			
Diuretics	Furosemide	C03CA01	2 (0.15)	
	Mannitol	B05BC01	6 (0.45)	
	Spironolactone	C03DA01	02 (0.15)	
IV Fluids	Hypertonic Solutions	B05DB	02 (0.15)	
	Normal saline	B05CB02	66 (5.03)	
	Ringer Lactate	B05BB01	15 (1.14)	
Total	83 (6.33)			
Minerals /Vitamins	Calcium	A12A	16 (1.22)	
	Multi-Vitamins	A11	06 (0.45)	
	Thiamine	A11DA01	5 (0.38)	
	Vitamin K	B02BA	01 (0.07)	
Total	28 (2.13)			
Steroids	Dexamethasone	H02AB02	21 (1.60)	
	Hydrocortisone	H02AB09	16 (1.22)	
	Prednisolone	H02AB06	11 (0.83)	
	Progesterone	G03DA03	03 (0.22)	
Total	51 (3.89)			
